# A molecular inversion probe assay for detecting alternative splicing

**DOI:** 10.1186/1471-2164-11-712

**Published:** 2010-12-17

**Authors:** Shengrong Lin, Wenyi Wang, Curtis Palm, Ronald W Davis, Kara Juneau

**Affiliations:** 1 Stanford Genome Technology Center, Department of Biochemistry, Stanford University School of Medicine, Palo Alto, CA USA; 2 Illumina Inc. 9885 Towne Centre Dr. San Diego, CA 92121, USA; 3 Department of Bioinformatics and Computational Biology, Division of Quantitative Sciences, The University of Texas MD Anderson Cancer Center, 1400 Pressler St. Unit 1410, Houston, TX 77030, USA

## Abstract

**Absract:**

**Background**

A sensitive, high-throughput method for monitoring pre-mRNA splicing on a genomic scale is needed to understand the spectrum of alternatively spliced mRNA in human cells.

**Results**

We adapted Molecular Inversion Probes (MIPs), a padlock-probe based technology, for the multiplexed capture and quantitation of individual splice events in human tissues. Individual MIP capture probes can be quantified using either DNA microarrays or high-throughput sequencing, which permits independent assessment of each spliced junction. Using our methodology we successfully identified 100% of our positive controls and showed that there is a strong correlation between the data from our alternative splicing MIP (asMIP) assay and quantitative PCR.

**Conclusion**

The asMIP assay provides a sensitive, accurate and multiplexed means for measuring pre-mRNA splicing. Fully optimized, we estimate that the assay could accommodate a throughput of greater than 20,000 splice junctions in a single reaction. This would represent a significant improvement over existing technologies.

## Background

During alternative splicing a single gene transcript is processed into several discrete mRNA isoforms, each of which contains different exonic sequences and can code for distinct proteins. These genetically related gene products can have diverse, sometimes antagonistic cellular functions (see *BCL2L1 *as an example [1]). Mis-splicing has been implicated in numerous human diseases [2-4] and therefore knowing the alternative splicing landscape of human tissue provides a starting point for evaluating splicing events as diagnostic markers for disease.

We wanted to develop an improved high-throughput method to precisely map the splicing events that dictate the spatial and temporal transcriptional content of an organism. Some low-throughput technologies that have worked for small numbers of genes include: reverse transcription polymerase chain reaction (RT-PCR), northern blots, real-time or quantitative PCR (qPCR) [5], the TaqMan assay [6], and Sanger sequencing. These technologies are effective for individual genes, but do not scale to whole-transcriptome analyses. DNA microarrays have been used to detect alternative splicing on a genomic scale, but data analysis has proven challenging due to their small dynamic range (less than 100-fold) [7] and high degree of cross-hybridization; related exon-exon junctions can have greater than 50% sequence similarity, making unambiguous assignment of each probe difficult [8,9]. To overcome these challenges researchers will typically assay different classes of tissues (e.g. brain versus non-brain) instead of individual tissues making single sample analysis difficult or impractical [9-11]. *De novo *high-throughput sequencing (HTS) is by far the most promising new technology for detecting novel splice sites [12,13]. The drawbacks of using whole transcriptome HTS to characterize alternative splicing are currently expense and limited dynamic range; the dynamic range is decreased for splicing studies because of the predominance of uninformative sequence, which overwhelms the small amount of alternatively spliced exon junction data obtainable. High-throughput sequencing would be much more effective if the sequences of interest could be captured prior to sequencing. Molecular Inversion Probes (MIPs) provide this exact utility; they quantitatively capture only the nucleic acid sequences of interest, which decreases cost and increases the dynamic range of individual sequencing runs.

**Figure 1:**
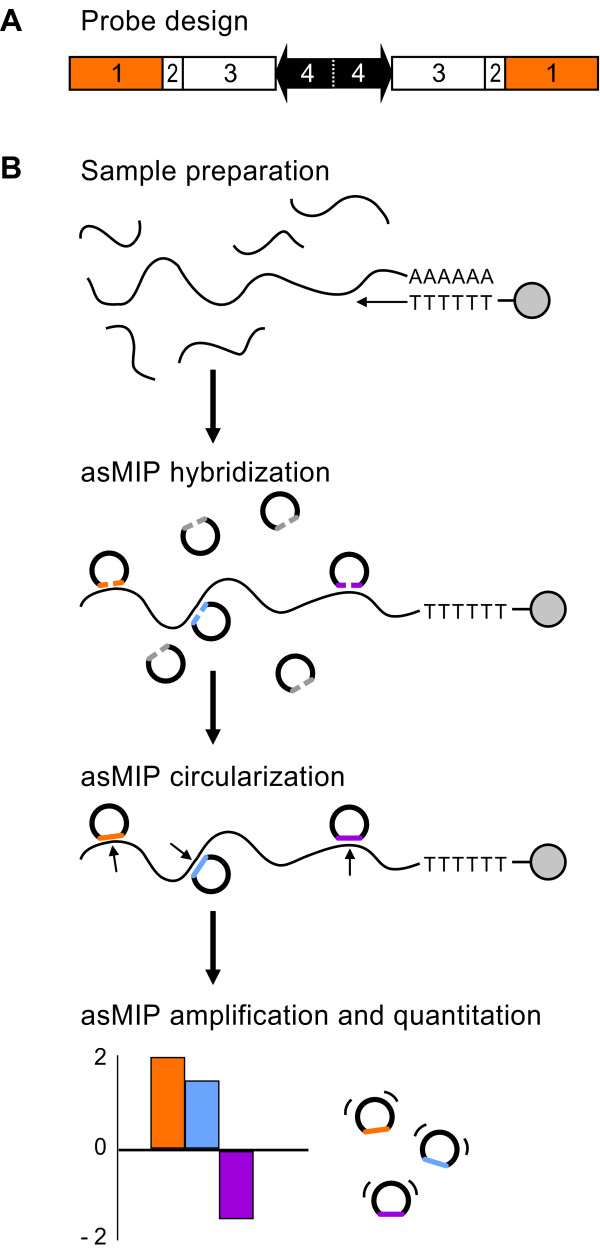
**A schematic of the asMIP methodology**. (A) The asMIP design. Unreacted probes terminate with 3'- and 5'-interrogation sequences (no. 1) abutted by two DraI cleavage sites (no. 2). Each probe contains two unique sequence tags (no. 3) and two common primer binding sites (no. 4). (B) The asMIP assay. Step 1, sample preparation: immobilized cDNA was reverse transcribed (arrow) from polyadenylated RNA (curved lines followed by AAAAAA) using oligo-dT primers (TTTTT), which were covalently attached to magnetic beads (gray circles). Following cDNA synthesis, RNA is digested and washed away. Step 2, asMIP hybridization: unreacted asMIP probes (flattened nicked circles) terminate with 3'- and 5'-interrogation sequences (gray and colored lines), which are homologous to the exon sequences that flank splice junctions on the cDNA. Probes quantitatively anneal to the appropriate exon-exon junction (colored probes). Probes that do not hybridize (gray probes) are washed away. Step 3, asMIP circularization: bound asMIPs are ligated into circles (small arrows). Step 4, asMIP amplification and quantitation: only the successfully ligated probes (contiguous circles) can be exponentially amplified using the common PCR primers (thin black lines). Each probe contains two unique sequence tags, which are amplified by PCR for multiplexed detection via array hybridization or high-throughput sequencing (bar graph).

MIPs are long, single stranded oligonucleotides that contain two interrogation sequences at their termini, one or two sequence tags for parallel readout with either microarrays or high-throughput sequencing, and two amplification primers that flank the sequence tags and are common to all MIPs (Figure 1). The MIP assay is performed in three steps: hybridization, circularization and amplification. During the MIP assay, probes hybridize to either side of a specific nucleic acid target sequence and bound MIPs are selected by circularizing the probes via ligation and amplified using common primers. MIPs can then be quantified using microarrays or HTS. The MIP technology was pioneered in our laboratory [14,15] and has been used to measure gene copy number in colorectal carcinoma [16] and quantify allele frequency in 56 different human cell lines [17]. We have now applied this technology to the analysis of alternative splicing.

Another sequencing capture strategy, which has been used to study alternative splicing, is cDNA-mediated annealing, selection, extension and ligation (DASL) [18]. DASL is very similar to the MIP alternative splicing strategy we developed, but with an important difference; interrogation sequences, barcodes and amplification sites are contained on two separate DASL probes whereas MIPs include all these components on a single probe. Thus, significantly higher concentrations of DASL probes are necessary for corresponding probes to find each other. Subsequently, cross-hybridization is an issue and multiplexing is limited to approximately 1500 probe-pairs [18]. In comparison, MIPs are effective at orders of magnitude lower concentration because the hybridization of the first interrogation sequence accelerates the hybridization of the second in a unimolecular reaction and cross-hybridization between MIPs is negligible. Thus, MIPs have been successfully multiplexed in sets of nearly 40,000 [19] and it is predicted that 100,000plex reactions are feasible [15].

This paper describes the development of a MIP-based assay for the high-throughput detection of alternative splicing events. The MIPs can accurately and quantitatively measure alternative splicing events for a number of genes in a variety of tissues. We looked at 208 exon junctions in 17 genes across five tissues, a total of 1040 splice events (208 × 5 = 1040). We showed that individual alternative splicing MIP (asMIP) measurements correlate well with direct qPCR measurements, as well as provide a concordance index (c) of 0.96 when compared with a set of known splicing controls. Moreover, we correctly identified tissue-specific splicing for 100% of the alternatively spliced control exons we assayed. In summary, we successfully developed a novel, high-throughput asMIP assay that can efficiently detect and quantify alternatively spliced exon junctions in a variety of tissues.

## Results

### Developing and testing an alternative splicing MIP assay

We set out to develop a MIP-based assay that could measure exon-exon junctions quantitatively and identify alternative splicing events. To obtain this level of sensitivity we customized the design of the MIP probes and optimized a rigorous four-step protocol: sample preparation, asMIP hybridization, circularization, and amplification (Figure 1).

There are four main differences between our single-stranded, DNA-based, alternative splicing MIPs (asMIPs) and the original single-nucleotide polymorphism (SNP) MIPs: 1) we included two sequence tags, 2) there is no gap between the probe termini when accurately annealed, 3) we did not include any deoxyuridine nucleotides between the amplification primers [14] and 4) we synthesized the target cDNA with primers that were covalently attached to magnetic beads; this allowed for extensive washing between enzymatic reactions, which likely improved quantitation by decreasing the background.

Seventeen genes, including 208 total exon-exon junctions, were selected to test the new asMIP assay. We identified 11 candidate genes that splice differentially in neural tissue [10], three genes that express distinct isoforms in smooth and skeletal muscle (*TPM1*, *TPM2*, and *TPM3*) [20] and three housekeeping genes (*ACTB*, *GAPDH*, *TUBA1B*), which served as loading controls (Table 1). In total, our set of known positive alternative splicing controls contained 14 genes, 25 exon-skipping events and 58 exon-exon junctions.

**Table 1:** asMIP targets

Gene	tissue specificity	**con**^**1**^	**alt**^**2**^	total
CAMK2D*	neural	8 (4)	5 (5)	13 (9)
CLTB*	neural	3 (3)	3 (3)	6 (6)
EHBP1*	neural	8 (3)	4 (4)	12 (7)
ERC1*	neural	8 (4)	10 (10)	18 (14)
FEZ2*	neural	2 (2)	9 (9)	11 (11)
MARK4	neural	8	3	11
MINK1	neural	11	10	21
MLLT4	neural	9	10	19
MYH10	neural	15	8	23
MYO6	neural	10	6	16
TPD52	neural	3	6	9
TPM1	muscle	5	7	12
TPM2	muscle	5	6	11
TPM3	muscle	4	6	10
ACTB	control	5	0	5
GAPDH	control	8	0	8
TUBA1B	control	3	0	3

	totals:	115	93	208

Numbers in parentheses correspond to total junctions analyzed by qPCR.

^1 ^The number of predicted constitutive exon junctions targeted by asMIPs indentified from RefSeq [21].

^2 ^The number of potentially alternatively spliced exon junctions targeted identified from RefSeq [21], of which 58 were confirmed in the literature to be alternatively spliced in the tissues we studied [10,20].

* Genes that were assayed with both asMIPs and qPCR.

### Minimal probe concentrations are required for the MIP assay

Our goal was to develop a high-throughput alternative splicing assay. Since the level of multiplexing possible for a ligation-based splicing assay is inversely correlated with probe concentration, we needed to show that the assay was reproducible with very small amounts of asMIPs. In our standard assay we used 10 femtomoles (fmol) of each probe in a 30 μl assay (333 pM). We compared the array intensities from the standard 10 fmol reaction against reactions with probe concentrations of 1 fmol and 0.1 fmol. We obtained *R^2 ^*values of 0.98 and 0.94, respectively, demonstrating that we could decrease probe concentration 10- to 100-fold without diminishing data quality (Additional Files 1A and 1B). Since our pilot asMIP experiments were approximately 200plex, this suggests that without optimization, performing a 20,000plex quantitative asMIP assay would be straightforward.

### Probe effects are addressed using either a biological reference or statistically modeled baseline

We built two analysis methods that extract and score alternative splicing information from asMIP and qPCR exon-junction data. For both methods we show that splice scores are significantly smaller for constitutive junctions than alternatively spliced junctions, which provides a straightforward approach for identifying alternatively spliced junctions.

The two asMIP analysis methods we developed both account for the sequence-based probe effects that frequently confound inter-probe comparisons. One method uses a biological reference (*R*-score), which is particularly useful if there is a well-defined or biologically meaningful reference sample that can be used as a baseline to make informative comparisons across different experiments. This reference-based score does require prior knowledge of which junctions are constitutive and can be negatively impacted by poor annotation or a limited number of constitutive sites. Our second analysis method is not dependent upon the accurate identification of gene-specific constitutive junctions and it does not require a biological reference. Instead, we used the data across all the tested tissues to build an additive, model-based score (*M*-score) for each gene. Different sample sets will result in differently modeled baselines, which in turn will affect *M*-score interpretation in the same way that different biological reference samples would affect *R*-scores.

For both methods, constitutively spliced junctions should score near zero while alternatively spliced junctions should have scores that differ substantially, either positively or negatively, from zero. To test our methodology, we identified a collection of 99 negative control junctions; these junctions are predicted to be constitutively spliced given that they are present in every mRNA isoform curated by the Reference Sequence Database (RefSeq) [21]. We then compared the constitutive junctions to a set of 58 positive control junctions; these junctions are known to be alternatively spliced in the tissues we studied [10,20]. We found that when the absolute values of the *R*- and *M*-scores were plotted for the two sets of control junctions, they deviated significantly from each other (p-value < 0.001) regardless of which technology was used (qPCR, asMIP-sequencing and asMIP-arrays). As expected, the constitutively spliced negative controls clustered tightly near zero; in comparison, the junctions known to be alternatively spliced provided significantly higher absolute values (Additional File 2).

**Figure 2:**
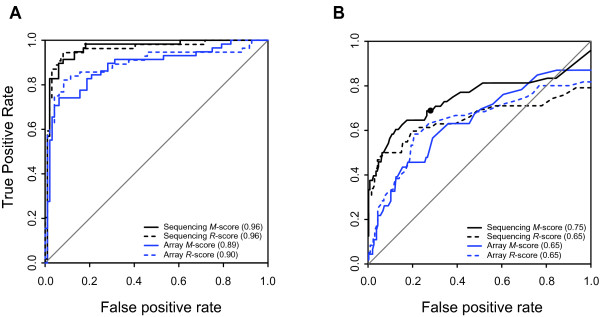
**The asMIP data correctly characterize splicing controls and correlate with qPCR measurements**. Receiver Operating Characteristic (ROC) curves comparing asMIP data with (A) junctions known from the literature to be alternatively spliced and (B) qPCR data. Data from asMIPs quantified using sequencing is black, asMIP data from arrays is blue; *R*-score data are dashed lines; *M*-score data are solid lines. (A) An analysis of 157 tissue specific splicing events: 58 positive alternative splicing controls [10,20] and 99 predicted constitutive negative controls [21]. For this plot, only relevant pairs of tissues, known to show alternative splicing for the genes assayed, were included in the calculation of *R*- and *M*-scores; skeletal and stomach data were used for tropomyosin genes, stomach and frontal lobe for brain specific genes, placenta and frontal lobe for ERC1. Absolute values of these scores were used. To generate the standard for comparison, positive spicing controls were assigned a value of 1, negative constitutive controls were 0. (B) An analysis of the 235 splicing events that were studied using both qPCR and asMIPs. For this plot we transformed ternary splice scores (1, 0, and -1) into binary scores (non-zero versus zero). Because there were cases where the qPCR and asMIP scores had the opposite sign, the ROC curves do not reach the upper right-hand corner of the graph. Individual ROC curves for positive or negative splice calls are located in Additional File 4. The solid black circle marks the point on the *M*-score sequencing line that corresponds to the +/-1.3 cutoff, which was used to identify alternative splicing.

Using receiver operating characteristic (ROC) curves we further evaluated the *R*- and *M*-score methodologies. Using the positive controls described above as our set of true-positives we calculated the areas under the curves (AUCs, equivalent to concordance index *c*) for all three technologies; positive controls were assigned a value of 1, negative controls were 0. The AUCs for the asMIP technology ranged between 0.89 and 0.96; AUCs for qPCR were 1.0 (Figure 2A and Table 2). We concluded that both *R*- and *M*-scoring strategies easily distinguished known alternative splicing events from constitutive ones.

**Table 2:** Evaluation of asMIPs using splicing controls and qPCR data

Statistic	*R*-Array	*R*-Seq	*M*-Array	*M*-Seq
AUC-controls^1^	0.90	0.96	0.89	0.96
AUC-qPCR^2^	0.65	0.65	0.65	0.76
Corr_qPCR_-controls^3 ^(*ρ*)	0.84	0.86	0.63	0.82
Corr_qPCR_-total^2 ^(*ρ*)	0.44	0.64	0.29	0.59

The table includes results from both the *R*- and *M*-score analyses (*R- *and *M-*), as well as data from asMIPs quantified with either arrays (Array) or sequencing (Seq). AUC and Pearson correlation (Corr, *ρ*) values are reported.

^1 ^asMIP data was compared to tissue specific, splicing controls from the literature: 58 tissue specific, alternatively spliced junctions [10,20] and 99 predicted constitutive junctions [21,34].

^2 ^asMIP data was compared to the complete set of qPCR data, 235 tissue specific splice events were analyzed.

^3 ^asMIP data from the set of splicing controls was compared to qPCR data from the same set of controls [10,20,21,34], 34 tissue specific splice events were analyzed.

### The asMIP results are correlated with direct qPCR analysis

With our analyses in place, we compared 235 qPCR-derived splice scores with asMIP splice scores and found that the data sets were highly correlated. Additionally, we observed that the sequenced asMIP data combined with the *M*-score analysis provided the best results.

We found a positive correlation between the splice scores for asMIPs and qPCR (Table 2 and Additional File 3). Given how extensively the two methods, qPCR and asMIPs, differ we did not expect, nor did we see, a perfectly linear agreement, but the correlation we did see supported our conclusion that asMIPs quantitatively measure alternative splicing events when either analysis method (*R*-score or *M*-score) is used.

Using receiver operating characteristic (ROC) curves we further evaluated the performance of the asMIPs compared to qPCR (Figure 2B). To carry out this analysis we first transformed all quantitative qPCR splicing scores into ternary splicing calls by assigning positive and negative cutoffs to the data; splice scores above or below the positive and negative cutoffs for each data set would receive splice calls of +1 and -1 and be labeled as alternatively spliced, while scores between the cutoffs would have a splice call of zero and would be labeled constitutive. For qPCR, we used the mean plus and minus three standard deviations to obtain cutoffs for *R*- and *M*-scores (+/-5.7 and +/-2.7 respectively). To plot an ROC curve that combined both positive and negative splicing data, it was necessary to convert ternary splice scores (1, 0, and -1) into binary scores (non-zero versus zero); true positives necessarily had the same sign (plus or minus) in both data sets. There were some instances where the qPCR data had the opposite sign as the asMIP data and thus, the ROC curves did not reach the upper right-hand corner of the graph (Figure 2B). Separate ROC curves for positive and negative splicing data can be found in Additional File 4.

We chose to use the asMIP *M*-score sequencing data for the majority of our downstream biological analyses because, compared to the other three methods (*R*-score array, *R*-score sequencing, and *M*-score array), it provided the largest area under the curve (0.76). Splicing calls were specified from this data using a cutoff of +/-1.3, which provided a good balance between sensitivity and specificity (0.68 and 0.72 respectively).

### 100% of the tissue-specific exon-skipping controls were identified using asMIPs

Using the asMIP data, we were able to confirm the tissue-specific alternative splicing of all 25 skipped exon controls with two or more asMIPs.

**Figure 3:**
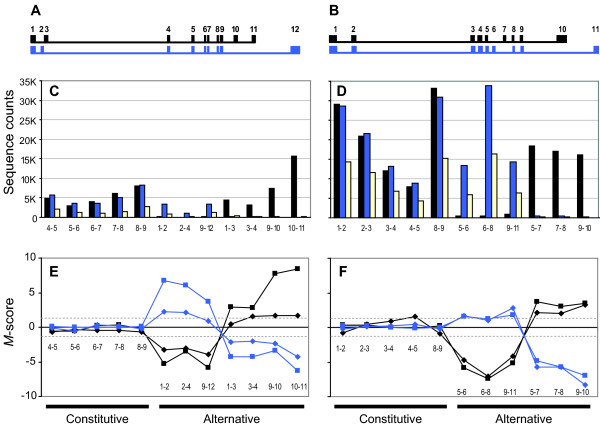
**Tissue-specific alternative splicing of *TPM1 *and *TPM2 *are confirmed using asMIPs**. Tissue-specific isoforms and splice-score data are color-coded; skeletal muscle is black, stomach is blue, and reference sample data is yellow. (A & B) Schematic representations of two muscle specific isoforms. Exons are represented by numbered boxes. Thin lines depict introns. (C & D) Graphs of the raw sequence counts for asMIPs interrogating the exon junctions specified (e.g. 4-5, 5-6 et cetera). Signal variation for constitutive junctions is due predominantly to sequence-based probe effects. (E & F) Graphs of the *M*-scores for each junction in which probe effects, gene expression differences and experimental variance have all been accounted for. The first five junctions listed for the gene are constitutively spliced and the remaining junctions are alternatively spliced. In (E & F) both the sequencing data (squares) and array data (diamonds) are presented for comparison. The gray dashed lines show the cutoff (+/-1.3) used to make splicing calls. The tissue-specific splicing of these control genes are clear; known constitutive junctions map near zero, alternatively spliced junctions all map above or below the cutoffs.

In this study we included three well-characterized tropomyosin genes that are frequently used as models for alternative splicing (*TPM1*, *2 *and *3*) [20]. These genes are known to have strong muscle-specific splicing patterns. Indeed, when we looked directly at the raw, un-normalized sequencing counts for asMIPs interrogating exon junctions in *TMP1 *and *2 *(Figures 3C &3D) and compared the results for skeletal muscle (black) with smooth stomach muscle (blue) it was apparent that smooth muscle exon junctions predominated in the smooth muscle tissue, while skeletal exon junctions predominated in the skeletal muscle. It is also evident from the raw data that the constitutive junctions, which might be expected to have similar counts, instead display large variations due to inherent sequence-based probe effects (Figures 3C &3D). Both the *R*- and *M*-scores account for these probe effects. Consequently, alternative splicing is better visualized in the *M*-score plots (Figures 3E &3F) where the constitutive junctions (the first five junctions plotted) score near zero, while the alternative junctions (last 6 or 7 junctions plotted) clearly score above and below the established cutoff (+/-1.3, gray dashed lines). Notably, we found that the sequenced asMIPs successfully identified the muscle specific splicing of all 19 exon junctions associated with the 11 skipped exons in all three tropomyosin genes tested.

**Figure 4:**
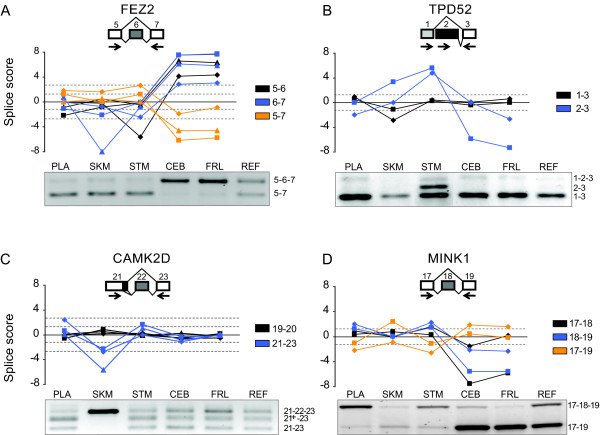
**Splice scores compare well with RT-PCR data**. Tissue-specific alternative splicing data are shown for four genes: *FEZ2 *(A), *TPD52 *(B), *CAMK2D *(C) and *MINK1 *(D). In each panel schematic representations of the analyzed splice junctions are shown on top. Exons are depicted as boxes and numbered; lines connecting the exons specify exon junctions; arrows show the position of PCR primers used for the RT-PCR gel electrophoresis splicing analysis. Below the schematics are graphs that plot splice scores for three sets of data: sequenced asMIPs (squares), array quantified asMIPs (diamonds) and qPCR (triangles). The *M*-score data are plotted for five different tissues: placenta (PLA), skeletal muscle (SKM), stomach (STM), cerebellum (CEB) and frontal lobe (FRL). Analyzed exon junctions are color-coded and specified to the right of each graph (e.g. "5-6" corresponds to data for the junction between exons five and six). Gray dashed lines show the *M*-score cutoffs for qPCR (+/-2.7) and asMIP data (+/-1.3). Tissue-specific RT-PCR, gel electrophoresis data is pictured below each splice-score graph. PCR primers surrounding the analyzed exon junctions were used to amplify cDNA reverse transcribed from RNA extracted from the specified tissues. A reference sample (REF) containing reverse-transcribed total RNA from >20 human tissues is pictured to provide a pictorial approximation of baseline splicing for comparison with the graph. The exons contained within each PCR gel band are specified to the right of each gel.

We next looked at our neural-specific splicing controls by comparing our asMIP *M*-score from sequence data against new or previously published gel electrophoresis data [10]. The splicing of exon-6 in *FEZ2 *is shown in detail as an example (Figure 4A). To evaluate the neural-specific splicing controls, we compared two tissues, stomach and frontal lobe, then tallied the number of alternatively spliced exon junctions that were observed. These two tissues were chosen because they best represented the splicing differences seen for non-neural versus neural-specific tissues when assayed by gel electrophoresis [10]. In one case, *ERC1*, the expression levels of the gene in stomach and skeletal muscle were too low to obtain asMIP measurements, thus, placenta data were used. For the neural-specific genes we identified 36/39 alternatively spliced junctions and were able to confirm all 13 exon-skipping events in 11 genes. The three missed junctions bridged the skipped exons from three genes: *FEZ2*, *MLLT4*, and *MYO6*. Interestingly, in all three genes where this occurred the short isoform was expressed well in all tissues while the long isoform was only expressed appreciably in the two neural tissues [10]. Thus, the failure to detect alternative splicing of the three bridging junctions may not indicate a technological error, but instead reflect the physiological isoform expression differences between these tissues.

### The asMIPs are capable of identifying splice events in individual tissues, as well as previously uncharacterized alternative splicing events

Using asMIPs quantified by sequencing and analyzed using the additive model (*M*-score) we detected 249 tissue-specific alternative-splicing events (out of 1040 total). The majority of which (79%) occurred in our set of potentially alternatively spliced junctions; these junctions were expected to show some degree of alternative splicing since they mapped to unique isoforms in the RefSeq database [21]. We used qPCR and gel electrophoresis to test approximately 30% of the identified junctions. We show that our method is capable of identifying splicing events within individual tissues, as well as events that displayed complex splicing patterns across multiple tissues. To illustrate this we discuss three specific examples in detail: *TPD52*, *CAMK2D*, and *MINK1*.

Tumor protein D52 (*TPD52*) is a small vesicle-trafficking protein that has been shown to be over expressed in a variety of human cancers [22]. *TPD52 *is known to have three isoforms [21]. Isoform-2 (NCBI Accession NM_001025253.1), which lacks exon-2, has been found to be the predominant isoform in brain tissue [10,23]. Using our asMIP data we were able to verify the expression of brain-specific isoform-2 and extend the analysis further to show that isoform-1 (NCBI Accession NM_001025252.1), which lacks exons 1, 6, and 7, is highly expressed in stomach tissue (Figure 4B). These splicing results for *TPD52 *demonstrate that asMIPs can identify tissue-specific splicing within a single tissue; clustering of similar tissues prior to analysis, as is commonly done for array data [9-11] is unnecessary.

Calcium/calmodulin-dependent protein kinase II delta (*CAMK2D*) is a serine/threonine protein kinase and the regulated splicing of exons 14, 15 and 16 helps direct postnatal heart remodeling [24]. There are six *CAMK2D *isoforms reported by RefSeq [21] and a seventh that was identified by Clark *et al. *2007, which we also validated as being neural specific. The neural-specific isoform lacks exon 14, but contains exons 15 and 16. Our asMIP studies on *CAMK2D *also demonstrated that isoform-3 (NCBI Accession NM_0012211.3), which contains exon 22, was preferentially expressed in skeletal muscle (Figure 4C); again implicating the involvement of *CAMK2D *in muscle function. Interestingly, this junction was initially mis-labeled in our dataset as being constitutive, but the asMIP data along with the *M*-score analysis was able to correctly identify it as being alternatively spliced (Figure 4C). This result exemplifies how a single asMIP in a single tissue can provide valuable splicing information even when the junction is thought to be constitutive.

Misshapen-like kinase 1 (*MINK1*) encodes a serine/threonine kinase that is involved in cytoskeleton reorganization, cell adhesion and cell motility [25]. *MINK1 *is alternatively spliced into four different isoforms [21]. In neural tissue, we verified that exon 21 was preferentially included in the mRNA [10] (not shown) and showed that exon 18 was excluded (Figure 4D), along with a shortened version of exon 16 (not shown). Only one of the four isoforms contains these specific exonic sequences, suggesting that a single isoform predominates in neural tissue, isoform-2 (NCBI NM_170663.3). We were also able to identify the single isoform (isoform-4 (NCBI Accession NM_001024937.2)) that is most highly expressed in stomach tissue by matching asMIP data with known isoforms; in stomach, exon 21 is preferentially excluded while exon 18 and the short form of exon 16 are included. The *MINK1 *gene illustrates how asMIPs can identify relevant splicing events within complex tissue-specific patterns of alternative splicing.

Our detailed analysis revealed that the asMIPs not only confirmed the alternative splicing of our controls, but also extended what was known regarding the tissue specificity of individual isoforms.

### High-throughput sequencing appears to outperform arrays for asMIP quantitation

We quantified our pools of reacted asMIPs either with DNA microarrays or HTS. Our results consistently showed that HTS outperformed arrays for quantifying asMIPs. For example, AUC and correlation values were significantly better (p-values < 0.05) for asMIPs quantified by HTS compared to array quantitation in three out of four analyses (Figure 2, Additional File 3 and Table 2). In addition, more of our positive splicing controls were identified when asMIPs were sequenced (100%) than when hybridized to arrays (96%). These data all suggest that the asMIP assay is more accurate when probes are quantified with high-throughput sequencing although arrays do perform adequately and could provide a more cost effective means of quantifying MIPs for some research projects.

## Discussion

In summary, we have developed a novel MIP-based alternative splicing assay that is sensitive, specific, and can be highly multiplexed. We compared our asMIP and qPCR methodologies and found that the correlation between asMIP and qPCR is good; for sequenced asMIP *M*-scores the Pearson correlation coefficient was 0.59 or 0.82 depending on the data set (Table 2) and the AUC was 0.76 (Figure 2B). Furthermore, when we looked at a set of splicing controls from the literature, sequenced asMIPs provided an AUC value of 0.96 (Figure 2A) and successfully identified 100% of the known, alternatively spliced exons along with 93% of their related junctions (for examples see Figure 3 and 4A). Additionally, several previously uncharacterized, tissue-specific splice events were revealed (Figures 4B-D). We have also shown that asMIP probes can be diluted to sub-femtomolar quantities (Additional File 1B). We conclude that the asMIP assay is capable of accurate, multiplexed quantitation of alternative splicing in human tissues; our data suggests that 20,000plex reactions will be feasible in the near future.

The advantage of using a sequence capture strategy to analyze alternative splicing in samples that could, in theory, be quantified directly using high-throughput sequencing is that you can dramatically increase the dynamic range of sequencing runs by increasing the amount of usable data. For example, if one looked at 20,000 exon junctions captured by asMIPs with 20-million short sequence reads, the dynamic range would be three orders of magnitude (10^3^). If one directly sequenced the sample with 20-million short reads mapped to the genome, only about 4% of those reads would likely overlap any exon junction (800,000 reads) [13]. If there are approximately 22,000 genes in the human genome with an average of 9-10 junctions (totaling ~200,000 junctions) [26] the dynamic range drops to 4 (800,000/200,000 = 4), less than one order of magnitude for direct sequencing.

One major advantage of asMIPs over other parallel sequence capture technologies such as DASL [18] is high level of multiplexing that is possible with unimolecular probes. Highly multiplexed reactions require small amounts of probe and we showed that only minute amounts of each asMIP are necessary for quantitative alternative splicing measurements; asMIP reactions carried out with 100 attomole (amol) of each probe were tightly correlated (*R^2 ^*= 0.94) (Additional File 1B). SNP MIPs have already been successfully used in ~40,000plex reactions [19] and 100,000plex reactions have been proposed [15]. The actual limit of multiplexing for MIPs has not been determined; it could, in practice, be significantly greater than 100,000plex.

Exon arrays also provide a high level of multiplexing, but the large variation in probe hybridization [8-11] and the smaller dynamic range [7] often confounds data analysis and impedes the identification of individual tissue specific splicing changes. The asMIP assay does not appear to suffer from the same limitations and consequently, can accurately identify independent tissue-specific splicing changes, like those seen for *TPD52 *and *CAMK2D *(Figures 4B &4C), whereas exon arrays struggle with the same task [9-11].

The asMIPs are well suited for quantifying known alternative splice events accurately in a single tube using a minimum of sample, but there are projects for which the technology is not ideal; large-scale analyses on a small number of samples might not be cost-effective and novel splice sites can not be identified using this method. Certainly, economies of scale would offset the initial financial outlay for large libraries of oligonucleotide probes and thus, asMIP collections would be a valuable resource, easily shared among laboratories. However, researchers conducting global splicing studies on only a few samples will likely find HTS more attractive than asMIPs, particularly when a large dynamic range is not essential. Similarly, researchers desiring to map isoforms or identify a collection of potential cancer biomarkers *de novo *would likely use high-throughput sequencing. But once those biomarkers have been identified then asMIPs are perfectly poised to accurately and cost-effectively characterize those splicing biomarkers in patient samples. Indeed, by barcoding each asMIP reaction prior to quantitation one could assay 1000 splice junctions in 20 samples using a single HTS reaction, producing a >1000-fold dynamic range. In this case, employing asMIPs would be less expensive and provide a larger dynamic range than carrying out 20 separate HTS reactions.

## Conclusions

We conclude that our asMIP technology can effectively quantify alternative splicing in human tissues and is well adapted for in-depth splicing studies on several hundred to tens-of-thousands of biologically important splice junctions in a variety of organisms.

## Methods

### MIP synthesis and design

Molecular Inversion Probes (MIPs) are single stranded DNA oligonucleotides with lengths ranging between 130 and 150 bases, synthesized in-house, using standard methods. Probes were used without purification at a cost of approximately $0.08 per base. For large scale studies it may be advisable to synthesize the oligos on DNA microarrays prior to bulk amplification via PCR [27] and subsequent transformation of the amplicons to single-stranded probes [28]. The architecture of the alternative splicing MIPs (asMIPs) differ only slightly from the original SNP MIPs (Figure 1A) [14]; the asMIPs contain an additional sequence tag (two tags total). The arrangement of specific sequences along the asMIP probe, in order from five-prime to three-prime, are as follows: 1) 5'-exon-junction interrogation sequence, 2) DraI cleavage site, 3) first sequence tag, 4) reverse PCR primer binding site GTGGTCTATG TCGTCGTTCG, 5) forward PCR primer binding site CGCTTTAGGT GCAGACACAA, 6) DraI cleavage site, 7) second sequence tag, 8) 3'-exon-junction interrogation sequence. Interrogation sequences were targeted to cDNA sequences that flank exon-exon splice junctions; the lengths of the interrogation sequences were adjusted so that each T_m _(melting temperature) would be near 60°C [29]. Sequence tags were 20-bases long and complementary to sequences contained on the TAG4 DNA microarray [30].

### Synthesis of immobilized cDNA

Human total RNA was obtained for five individual tissues and one tissue mixture from Clontech: placenta (catalog no. 636527), skeletal muscle (catalog no. 636534), stomach (catalog no. 636578), cerebellum (catalog no. 636535), frontal lobe (catalog no. 636563), and Human Universal Reference Total RNA (REF) (catalog no. 636538).

Immobilized cDNA was synthesized on Dynabeads Oligo (dT)_25 _(Invitrogen catalog no. 61002) according to the manual. Briefly, polyA+ mRNA was purified from 1 μg of total RNA (Clontech) that was mixed with 5 μl of Dynabeads in 20 μl binding buffer (20 mM Tris-HCl pH 8.0, 2 mM EDTA, 1 M LiCl). The beads were washed three times at room temperature in 50 μl of wash buffer (1 mM Tris-HCl pH8.0, 1 mM EDTA, 0.15 M LiCl ). Reverse transcription (RT) was carried out in 20 μl of RT mix (4 μl 5× RT buffer (Invitrogen, catalog no. 18080-044), 1 μl 0.1 M DTT, 1 μl SuperScript III (Invitrogen, catalog no. 18080-044), 1 μl 10 mM dNTPs, 13 ul water); RT reactions were incubated and rotated in UVP-HB-500 Mini Hybridization Oven (UVP, Upland, CA, USA) at 50°C for 30 minutes and then at 55°C for an additional 30 minutes. RNA was removed by incubating the completed cDNA synthesis reaction with 5U RNase H. Immobilized cDNA was washed and resuspended in 10 μl washing buffer.

### MIP selection: target hybridization and probe circularization

The asMIP reaction is carried out in three parts: probe hybridization, circularization, and signal amplification/quantitation. A multiplexed pool of 208 asMIP oligos, each at a concentration of 0.5 nM, was used for the assay.

#### Target hybridization

Each asMIP reaction required 20 μl of the asMIP pool; the pool was denatured in 1× Ampligase buffer (Epicenter, catalog no. A3202k) at 95°C for 5 minutes, then chilled on ice before being added to the prepared immobilized cDNA (10 μl). Hybridization of the MIPs to the cDNA took place in three steps: the mixture was incubated at 70°C for 10 minutes, slowly cooled at ~1 degree per minute to a final temperature of 58°C, then held at 58°C for another two hours. After incubation, the annealing reaction was washed three times with Dynabead wash buffer, twice at 58°C and once at room temperature.

#### Probe circularization

The wash buffer was removed from the beads and 10 μl of ligation mix (1 μl Ampligase, 1 μl 10× ligation buffer and 8 μl water) was added; the ligation reaction was incubated at 58°C for 30 minutes. Following ligation, the beads were washed three times with Dynabead wash buffer: twice at 58°C and once at room temperature. The wash buffer was removed and beads were resuspended in 40 μl of TE (10 mM Tris, 1 mM EDTA, pH 8.0). Circularized MIPs were dissociated from the cDNA by holding the sample at 95°C for 5 minutes and chilled on ice. The ligated MIPs were further purified and fully eluted from the immobilized cDNA by digesting all linear DNA with exonucleases; 4 μl of exonuclease mix (3.5 μl 10× Exonuclease Buffer, 0.5 μl Exo I (NEB catalog no. M0293S. 1 μl Exo III (NEB catalog no. M0206S)) was added to the MIP reaction and samples were incubated at 37°C for 45 minutes. The exonucleases were inactivated by heating samples to 94°C for 20 minutes.

### MIP amplification and labeling

#### Microarray sample preparation

The specific process used for amplifying circularized asMIPs was tailored to the method of quantitation; for arrays, MIPs were amplified with biotinylated PCR primers before being hybridized to microarrays. Specifically, 1 μl of eluted asMIPs were added to a 50 μl PCR reaction containing 5'-biotinylated P1 and P2 (P1: bio-CGAACGACGA CATAGACCAC, P2: bio-CGCTTTAGGT GCAGACACAA) primers at 0.6 μM concentration and 1× Platinum PCR SuperMix (Invitrogen, catalog no. 11306-016). PCR reactions were carried out using the following three-step thermal profile: denaturation at 94°C for 15 seconds, annealing at 60°C for 1 minute and extension at 72°C for 5 s. The resulting PCR products were treated with 20 u of DraI (NEB catalog no. R0129S) incubated at 37°C for 1 hour; DraI digestion was used to separate the two labeled sequence tags, as well as remove the now extraneous interrogation sequences, which could confound hybridization.

#### High-throughput sequencing asMIP sample preparation

Unique primers were used to amplify asMIPs quantified using Illumina HTS; in all other respects the PCR reaction conditions were identical to those specified for microarray sample preparation. The primers used for HTS contained: 1) the adaptor sequences used for cluster generation, as specified by Illumina (see underlined sequence below, 2) a two-nucleotide barcode (NN) that was sample specific so that seven tissue-specific samples could be quantified in a single sequencing channel and 3) the common primers contained within the asMIPs. Explicitly the sequences of the forward and reverse primers, respectively, are as follows: for sequencing Tag 1, AATGATACGG CGACCACCGA GATCTACACT CTTTCCCTAC ACGACGCTCT TCCGATCTNN CGACGACATA GACCAC and CAAGCAGAAG ACGGCATACG AGCTCTTCCG ATCTCGCTTT AGGTGCAGAC ACAA; for sequencing Tag 2 AATGATACGG CGACCACCGA GATCTACACTC TTTCCCTACA CGACGCTCTT CCGATCTNNT TAGGTGCAGA CACAA and CAAGCAGAAG ACGGCATACG AGCTCTTCCG ATCTCGAACG ACGACATAGA CCAC.

### Microarray hybridization and quantitation

Genflex Tag 16K Array v2 chips (Affymetrix, catalog no. 511331) were pre-hybridized with 90 ul 1× hybridization buffer (100 mM MES pH 6.6, 1 M NaCl, 20 mM EDTA, 0.01% Tween 20) for 15 minutes at room temperature. After removal of the hybridization buffer, 0.2, 2.0 or 20 μl of DraI-cut, biotinylated asMIP sample was hybridized to the array in a sample buffer that contained: 1× hybridization buffer, 1× Denhardt's (Sigma-Aldrich, catalog no. 30915), 1 pmol/μl anti-sense oligonucleotides that were reverse complementary to forward and reverse PCR primers, and 0.6 fmol/μl B213 control oligonucleotide (bio-5'-CTGAACGGTAGCATCTTGAC-3'). The linear dynamic range of the arrays is limited to two orders of magnitude (100-fold) (Additional File 1C). For this assay we needed to increase the linear dynamic range of the data we collected so we hybridized three volumes of biotinylated asMIP sample (0.2, 2.0 or 20 μl) for each tissue type. This effectively increased the dynamic range by 100-fold to approximately four orders of magnitude. Arrays were washed and stained using a GeneChip fluidics station 450 (Affymetrix, Santa Clara, CA, USA) and scanned on a GeneChip scanner 7G (Affymetrix, Santa Clara, CA, USA). Fluorescent data were extracted using the GeneChip operating software version 1.4 provided by Affymetrix.

### High-throughput sequencing

Purified asMIP ligation products were amplified with modified sequencing primers (described above). Amplification products were analyzed by gel electrophoresis and the products from six tissue samples (placenta, skeletal muscle, stomach, cerebellum, frontal lobe, and the universal reference) were combined for parallel sequencing. Molecular Probes PicoGreen DNA quantitation kit (Molecular Probes, catalog, no. P-7589) was used to precisely quantify the combined MIP products, 1.4 ng of which was used for sequencing on the Illumina Genome Analyzer (Illumina, San Diego, CA, USA). We obtained 1.9 million reads from a single lane of sequencing, which could be uniquely mapped to the asMIP sequence tags. Only perfect sequence matches were counted. The average coverage per tag was 984X, with a maximum coverage of 38,000X; 75% of all tags had more than 10 reads.

### Direct quantification of exon-exon junctions using quantitative-PCR (qPCR)

Two sets of primers were designed to interrogate each exon-exon junction for qPCR quantitation; the primer sets were designed such that one primer would hybridize across the exon-exon junction with exactly five 3'-nucleotides on the downstream side and the remaining 17+ nucleotides on the up-stream side of the junction. We designed two sets of primers for each junction; in one set the forward primer crossed the junction while the reverse primer was positioned <100 nucleotides downstream, in the other the reverse primer crossed the junction and the forward primer was <100 nucleotides upstream. The primers were designed to have melting temperatures above 60°C [29].

The cDNA samples were reverse transcribed from the same tissue-specific RNA used for the asMIP studies. We used the protocol and reagents provided in the SuperScript III Kit (Invitrogen, catalog no. 18080-044) to reverse transcribe 1 μg of tissue-specific total RNA (Clontech) in a 20 μl reaction containing 2.5 μM oligo dT(20) primer.

Quantitative PCR reactions contained 0.2 μl of the above reverse transcription reaction and 10 μl of a reaction mix prepared by pre-mixing 0.5 μM of both forward and reverse primers with 1× SYBR Green PCR Master Mix (Applied Biosystems, catalog no. 4309155). Using a 7900 HT real time PCR system (Applied Biosystems, Foster City, CA, USA) reactions were denatured for 10 minutes at 94°C, followed by a two-step amplification cycle: 94°C for 5 seconds then 60°C for 60 seconds. Data were collected during the 60°C incubation.

We used the SDS RQ Manager 1.2 software from Applied Biosystems to calculate a cycle threshold(Ct) for each primer set in six cDNA samples: five tissue-specific samples plus the Universal Reference mixture of tissues (REF). The relative tissue-specific expression level is defined as the difference in Ct values between two samples (ΔCt); the ΔCt for each primer set was calculated by subtracting the tissue-specific Ct value from the Universal Reference (e.g. for primer set 1, ΔCt^1 ^_liver _= Ct^1 ^_REF _- Ct^1 ^_liver_). As described above, each junction was interrogated by two sets of primers; if both primer sets worked, ΔCt data were averaged for the samples. If one of the two sets failed then the data were not combined and only the informative primer set was used in downstream calculations. In total, ΔCt data were collected for 47 exon-exon junctions in five individual human tissues, which provided data for 235 splice events (47 × 5 = 235). The qPCR data were used to test the alternative splicing identification methodology we developed for the asMIP array and sequencing data.

### Data processing used to identify alternative splicing events

The five main steps/analyses we used to test the asMIP assay and identify alternative splice are as follows:

1. Subtract background fluorescence signal (microarrays).

2. Identify and select data in dynamic range (microarrays).

3. Calculate normalized splice scores using a reference sample (*R*-score).

4. Calculate normalized splice scores using a statistical model (*M*-score).

5. Identify cutoffs for assigning alternative splicing events.

#### Background subtraction

The background was defined as the average signal intensity across 20 unused features common to all TAG4 microarrays. Each raw data point was normalized by subtracting the background. After the background subtraction, we calculated the standard deviation (SD) of the 20 unused spots, data points having values at or below 1.96 times the SD were excluded from further analysis. This method of background subtraction was used for analyzing the microarray data, but was not necessary for analyzing the qPCR or HTS data.

#### Identify and select data in dynamic range

To increase the dynamic range of the microarrays by 100-fold we hybridized each tissue-specific asMIP sample to three arrays at three sample dilutions that differed by ten-fold (0.2, 2.0 or 20 μl of sample per array). The lower end of the dynamic range for each chip was equal to the background plus 1.96 × SD (described above). The upper end of the dynamic range was based upon informed observation and set at 3500 units. To identify the sample dilution (0.2, 2 or 20 μl) that provided the best array data for all the data points within a specific gene, we identified the sample dilutions having the most data points within the dynamic range. We did this simple analysis for each gene, in every tissue, at all three sample dilutions (0.2, 2 and 2.0 μl).

#### Calculate R-scores

We calculated a tissue-specific *R*-score (reference based score) for each junction in each tissue. To address technical probe effects for asMIPs resulting from sequence-related hybridization differences and enzymatic reaction biases we calculated

(1) $$d_{i}=\theta_{i}-\theta_{0}$$

where *θ *denotes log base 2 of array intensities or Illumina digital counts, *i *denotes tissue sample and *0 *denotes reference sample. Our reference sample is a commercially produced mixture of tissues from Clontech (Human Universal Reference Total RNA, catalog no. 636538). The log transformation was chosen for the sequencing data because we had numerous counts for each tag (median = 94) and the distribution of the data was right-skewed; we assumed and later verified that the probe effects were additive at the log scale.

To extract the splicing information from varying baselines of gene expression, for each candidate junction *j *in gene *g*, we calculated

(2) $$f_{j,k,i,g}=d_{j,k,i,g}-{\bar{d}}_{c,k,i,g}$$

where *c *denotes tissue-specific constitutive junction data (constitutive annotation based on BLAST [31] alignment of common isoforms), and *k *denotes tag measures (*1 *or *2*) for each junction. In order to normalize between genes, we averaged the *f*-measure over the two tags for each junction and then calculated our *R*-score as

(3) $$R_{j,i,g}=f_{j,i,g}/s_{g}$$

where *g *denotes genes, *s_g _*denotes the standard deviation of *f *calculated for all constitutive asMIPs for that gene. This procedure is performed using R (http://www.r-project.org/) function *rlm *for robust estimation. Normalizing for gene specific variation using *s_g _*was important when making inter-gene splicing rankings, which was necessary to plot the ROC curves in Figure 2 and Additional File 4. Calculating the *R*-scores for qPCR was identical to the calculations made for asMIP data once it was taken into consideration that output for qPCR (cycle threshold (Ct)) is already log_2 _transformed. For example, the qPCR log_2 _ratio of sample to reference was calculated by simply subtracting the Ct for the reference from the Ct of the sample and so forth.

#### Calculate M-scores

To build an analysis method that requires no prior knowledge of which junctions are alternatively spliced and to identify an alternative method to evaluate our technology using the pilot data, we applied an additive model to produce *M*-scores for each junction in each tissue. In brief, we have

(4) $$\theta_{j,k,i,g}=p_{j,k,g}+t_{i,g}+\varepsilon_{j,k,i,g}$$

where *θ *represents log base 2 of the raw signals, *j *denotes junctions, *i *denotes tissues, *g *denotes genes, *k *denotes tag measures (*1 *or *2*) for each junction, *p *denotes technical effects, *t *denotes baseline gene expression level, and *ε *denotes random errors with mean 0 and variance σ^2^. Our model is similar to the FIRMA model [32], previously developed for detecting differential alternative splicing using exon arrays. With this modeling strategy, we hope to robustly estimate and address in our current data both the technical effects, by averaging over all tissues for each junction (i.e. replace *θ_0 _*in equation (1) with ${\hat{p}}_{j,k,g}$) and the baseline of gene expression, by averaging over all junctions for each tissue and each gene (i.e. replace ${\bar{d}}_{c,i,g}$ in equation (2) with ${\hat{t}}_{i,g}$). The estimation of these parameters were performed using median polish [33], which fitted an additive model by alternatively removing the row (-junction) and column (-tissue) medians until the proportional reduction of the sum of absolute residuals is negligible [33]. The above described procedure provided us with

(5) $$f_{j,k,i,g}^{*}={\hat{\varepsilon }}_{j,k,i,g}$$

where f* is analogous to *f *in equation (2). Our final *M*-score is calculated as

(6) $$M_{j,i,g}=f_{j,i,g}^{*}/s_{g}^{*}$$

where s*_g _is the MAD of f* in all junctions and tissues in gene *g*. For more details please see our step-by-step example for calculating the *M*-scores for the *CLTB *gene (Additional File 5).

Following our analysis, we tested our assumption that the probe effects were additive by verifying the linearity and normality of our additive model with sequencing data. We found no significant deviations from these assumptions when there were at least four reads per tag (85% of all data).

We used only the known splice junction controls to generate Figure 2A, Additional File 2 and rows 1 and 3 in Table 2. We calculated *M*-scores and *R*-scores using just the pairs of tissues known to show alternative splicing for the genes assayed; skeletal and stomach data were used for tropomyosin genes, stomach and frontal lobe for brain specific genes, placenta and frontal lobe for ERC1. To plot the ROC curve in Figure 2A we used the absolute value of the splice score differences from each pair of tissues. These differences are also splice scores with the baseline expression shifted to one of the tissues in each pair. These tissue-restricted *M*- and *R*-scores were compared with the splicing controls. Constitutive splicing controls were assigned a value of 0; alternatively spliced controls were assigned 1.

#### Cutoff Identification

After calculating the *R*- and *M*-scores for all three sets of data (qPCR, arrayed asMIPs, and sequenced asMIPs) we identified cutoffs to categorize splicing events as alternatively or constitutively spliced in our data set. Firstly, we defined stringent cutoffs for the qPCR data in order to compile a list of true positives. Any scores that are significantly higher or lower than the boundary for the constitutive junctions may point to candidate alternatively spliced junctions. We chose a cutoff for the qPCR score that was equal to the mean (-0.13 *R*-score and -0.05 *M*-score) minus three standard deviations (5.55 *R*-score and 2.64 *M*-score) from the mean. The calculated cutoffs for *R*- and *M*-score qPCR data were +/-5.7 and +/-2.7 respectively; we assumed symmetry in the score for positive and negative changes. We used this cutoff to define a set of true positives for alternative splicing events, which we used to assess the asMIP results. The cutoff for the asMIP data was 1.3, which provided a good balance between sensitivity and specificity (0.68 and 0.72 respectively); to facilitate the comparison between array and sequencing quantitation we assigned the same cutoff for both asMIP readouts (arrays and HTS).

## List of abbreviations

MIP: Molecular Inversion Probe; asMIP: alternative splicing Molecular Inversion Probe; HTS: high-throughput sequencing; RT-PCR: reverse transcription polymerase chain reaction; qPCR: quantitative PCR; SNP: single-nucleotide polymorphism; SD: standard deviation; DASL: cDNA-mediated annealing, selection, extension and ligation; ROC: receiver operating characteristic; AUC: area under the curve; *R*-score: reference-based splicing score; *M*-score: model-based splicing score.

## Authors' contributions

SL designed, performed and analyzed experiments; WW devised and conducted the *R*-score and *M*-score splicing analyses, and assisted in writing the paper; CP constructed the asMIPs. RWD provided general advice; KJ initiated and managed the project, designed experiments, analyzed the data, and wrote the paper. All authors have read and approved the final manuscript.

## Supplementary Material

Additional file 1 **This figure shows the reproducibility of the asMIP assay using decreasing amounts of probe library (Figures A & B), and presents evidence that the linear dynamic range of the arrays used to quantify asMIPs is approximately 100-fold (Figure C)**. Click here for file

Additional file 2 **Data presented in this figure demonstrate that splice scores for alternatively spliced junctions are much larger than for constitutive junctions**. Click here for file

Additional file 3 **The plots in this figure show the correlation between splice scores derived from asMIP assays compared to qPCR**. Click here for file

Additional file 4 **This figure shows two Receiver Operating Characteristic (ROC) plots comparing qPCR splicing calls against asMIP splicing calls made at various cutoffs for either the positive (Figure A) or negative (Figure B) qPCR splicing calls**. Click here for file

Additional file 5 **A detailed, step-by-step example calculation for *M*-score for one gene**. Click here for file
