# Supplementary material for: A molecular inversion probe assay for detecting alternative splicing
Source: BMC Genomics. 2010 Dec 17;11:712. doi: 10.1186/1471-2164-11-712 (PMC3022918; doi:10.1186/1471-2164-11-712)
Supplement: Additional file 1 — This figure shows the reproducibility of the asMIP assay using decreasing amounts of probe library (Figures A & B), and presents evidence that the linear dynamic range of the arrays used to quantify asMIPs is approximately 100-fold (Figure C). [file 1471-2164-11-712-S1.PDF]

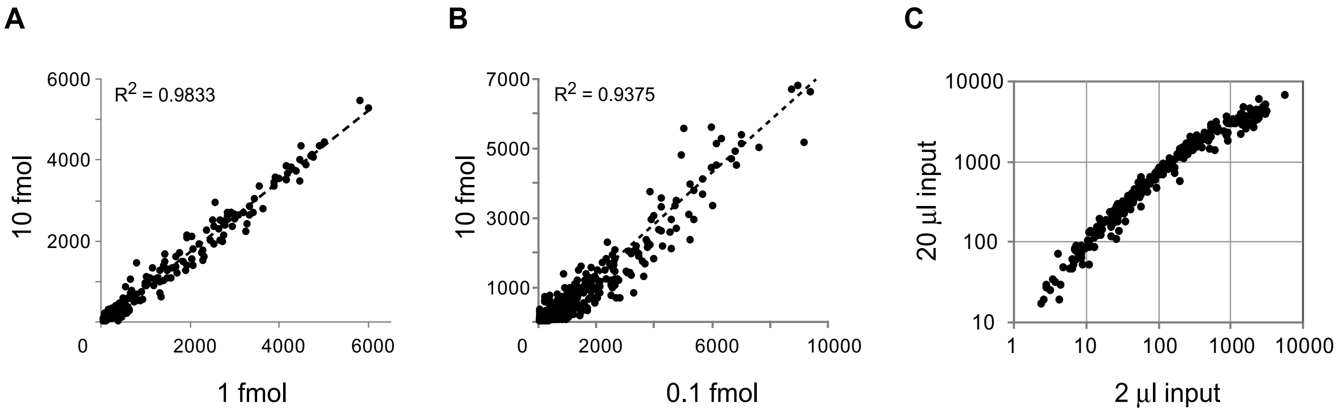

Additional File 1: Performance of asMIPs quantified with arrays. Array intensity values for reactions containing (A) 1 fmol of each asMIP or (B) 0.1 fmol are plotted on the x-axis while reactions containing 10 fmol are plotted on the y-axis. The four asMIP reactions shown were carried out on 1 µg of total RNA extracted from human placenta. (C) Shows that a single array provides a 100-fold dynamic range for quantifying asMIPs. Different amounts, 2 µl (x-axis) or 20 µl (y-axis), of a single completed asMIP reaction were hybridized to two separate arrays and array intensities are plotted on a logarithmic scale. The asMIP reaction was carried out on 1 µg of total RNA extracted from human placenta.
