# Supplementary material for: A molecular inversion probe assay for detecting alternative splicing
Source: BMC Genomics. 2010 Dec 17;11:712. doi: 10.1186/1471-2164-11-712 (PMC3022918; doi:10.1186/1471-2164-11-712)
Supplement: Additional file 2 — Data presented in this figure demonstrate that splice scores for alternatively spliced junctions are much larger than for constitutive junctions. [file 1471-2164-11-712-S2.PDF]

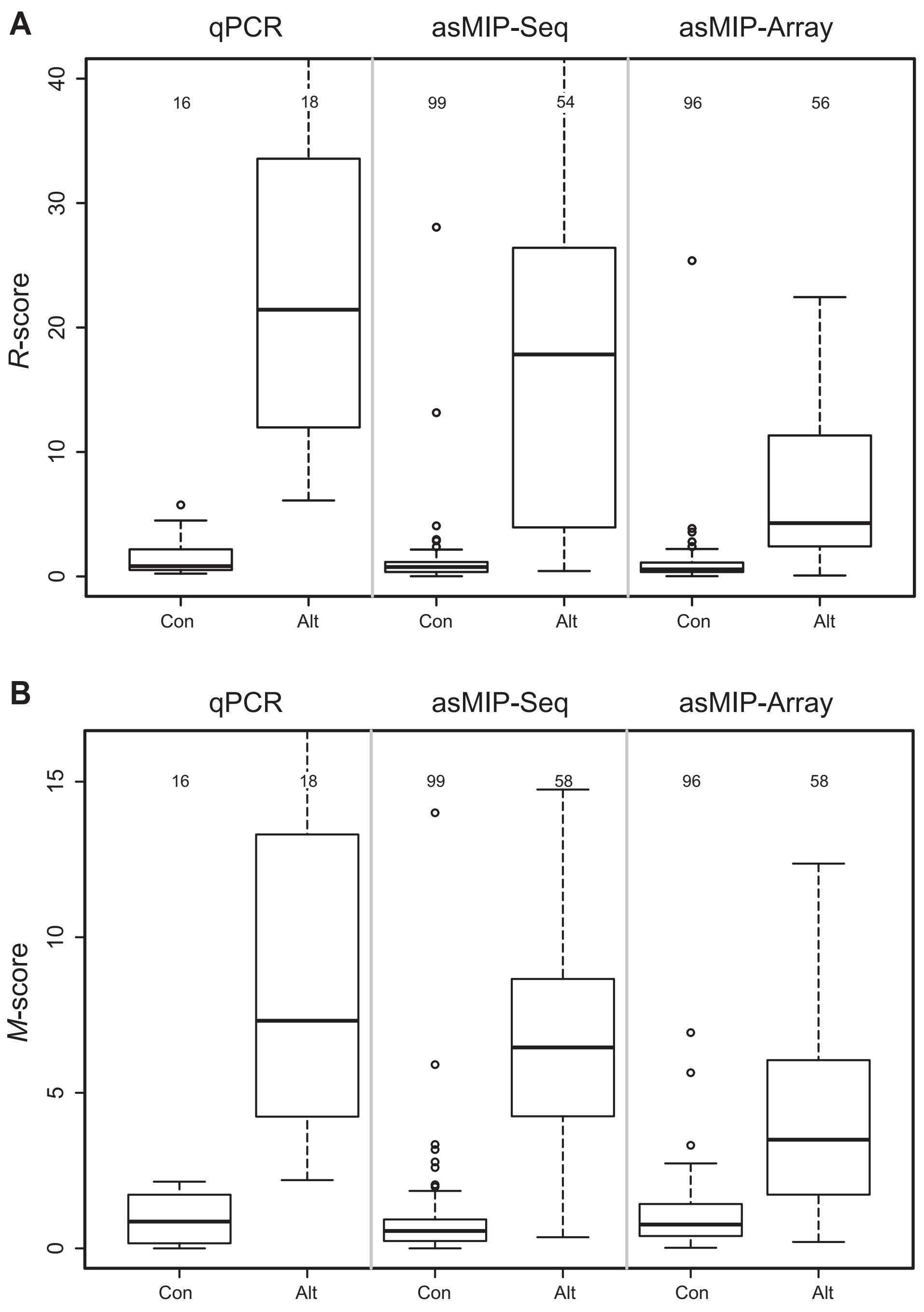


**Additional File 2:** Splice scores for alternative splicing events are much larger than for constitutive events. In these boxplots the absolute values of *R*- and *M*-scores (Figures A and B respectively) are plotted for our two sets of control junctions: 58 junctions known from the literature to be alternatively spliced (Alt) and 99 junctions predicted to be constitutive (Con); only 18 spliced and 16 constitutive control junctions were analyzed using qPCR. Data are presented for three different technologies: qPCR, sequenced asMIPs (asMIP-Seq), and array quantified asMIPs (asMIP-Array). These analyses were carried out on data from relevant tissue pairs known to display alternative splicing; skeletal and stomach data were used for tropomyosin genes, stomach and frontal lobe for brain specific genes, placenta and frontal lobe for ERC1. Within the boxplots, the lower bound, middle, and upper bound of the boxes represent the 25th percentile, median, and 75th percentile; outliers are plotted as open circles. The number of junctions available for each analysis is specified on top of the boxplots.
