# Supplementary material for: A molecular inversion probe assay for detecting alternative splicing
Source: BMC Genomics. 2010 Dec 17;11:712. doi: 10.1186/1471-2164-11-712 (PMC3022918; doi:10.1186/1471-2164-11-712)
Supplement: Additional file 3 — The plots in this figure show the correlation between splice scores derived from asMIP assays compared to qPCR. [file 1471-2164-11-712-S3.PDF]

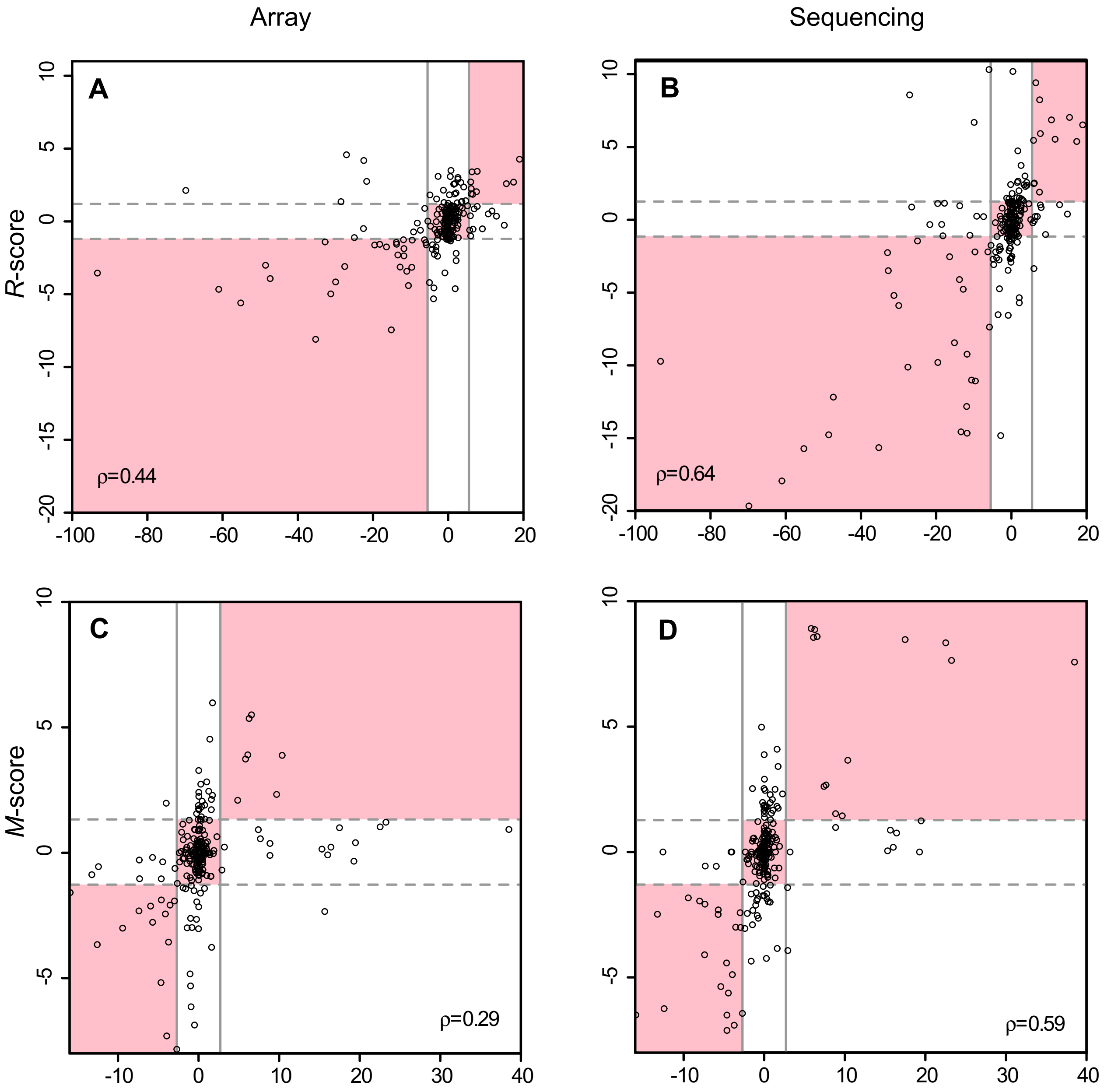

Additional File 3: Graphs show that the splice scores for asMIP (y-axes) and qPCR data (x-axes) are correlated. Each data point represents one junction being studied in one tissue. The cutoffs for making splicing calls are shown as dashed gray lines for the asMIP data (+/-1.3) and solid gray lines for the qPCR data (+/-2.7). Pink shaded boxes highlight data where the two technologies are in agreement; the middle box contains data that are between the two cutoffs signifying junctions that are not alternatively spliced; the lower left-hand and upper right-hand boxes contain data that both technologies have identified as being alternatively spliced. (A) Correlation of *R*-scores calculated using asMIP-array data (y-axis) and qPCR data (x-axis). (B) Correlation of *R*-scores calculated using asMIP-sequencing data (y-axis) and qPCR data (x-axis). (C) Correlation of *M*-scores calculated using asMIP-array data (y-axis) and qPCR data (x-axis). (D) Correlation of *M*-scores calculated using asMIP-sequencing data (y-axis) and qPCR data (x-axis).
