# Supplementary material for: A molecular inversion probe assay for detecting alternative splicing
Source: BMC Genomics. 2010 Dec 17;11:712. doi: 10.1186/1471-2164-11-712 (PMC3022918; doi:10.1186/1471-2164-11-712)
Supplement: Additional file 4 — This figure shows two Receiver Operating Characteristic (ROC) plots comparing qPCR splicing calls against asMIP splicing calls made at various cutoffs for either the positive (Figure A) or negative (Figure B) qPCR splicing calls. [file 1471-2164-11-712-S4.PDF]

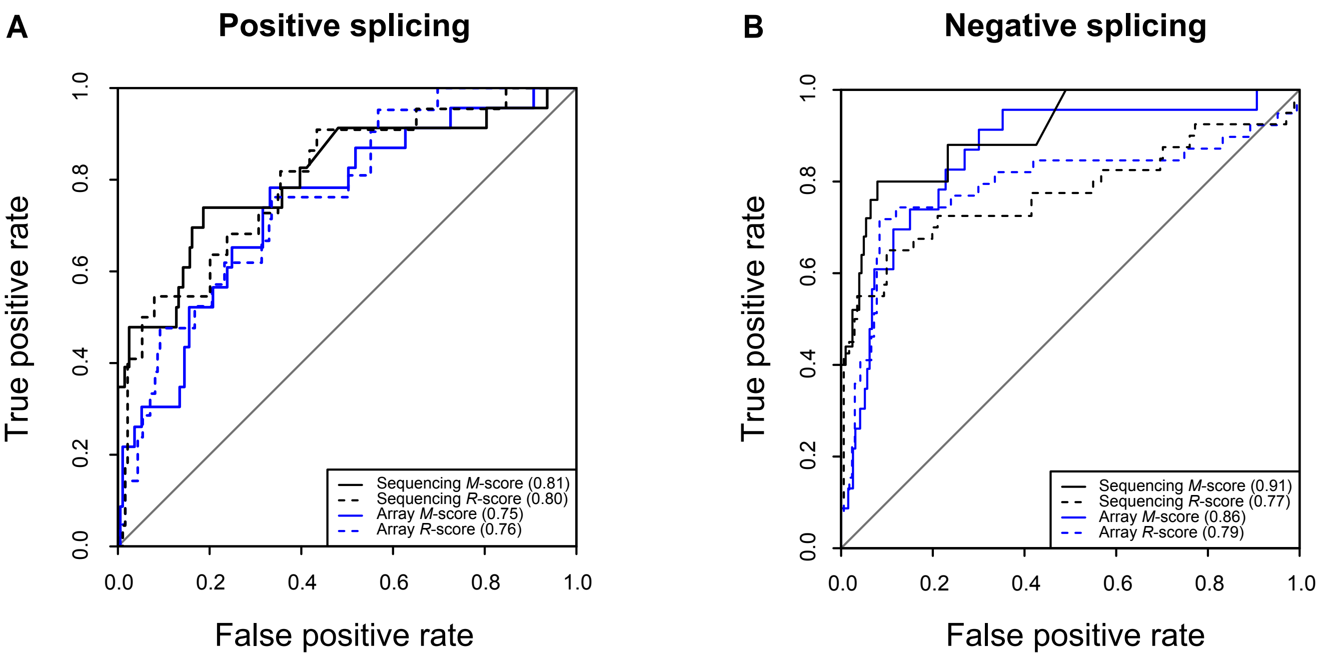

Additional File 4: This figure shows two Receiver Operating Characteristic (ROC) plots comparing qPCR splicing calls against asMIP splicing calls made at various cutoffs. Array data (blue) and sequencing data (black) has been analyzed by two different methods: *R*-scores (dashed lines) or *M*-scores (solid lines). For these two ROC plots the sets of (A) positive and (B) negative qPCR splicing calls were analyzed separately.
