# Supplementary material for: A molecular inversion probe assay for detecting alternative splicing
Source: BMC Genomics. 2010 Dec 17;11:712. doi: 10.1186/1471-2164-11-712 (PMC3022918; doi:10.1186/1471-2164-11-712)
Supplement: Additional file 5 — A detailed, step-by-step example calculation for M-score for one gene. [file 1471-2164-11-712-S5.PDF]

Example for the M-score Calculation

Here we provide a detailed example of calculating the M-score for all junctions in gene *CLTB*, in R environment (http://www.r-project.org), with the asMIP-seq data.

1. The raw sequencing count data is shown below. With each junction-tag in a row (12 rows in total: 6 junctions X 2 tags/junction) and each tissue in columns 5 to 9.

```
> dat.CLTB
```

	Gene	Exon_Junction	Junction_Type	TAG	PLA	SKM	STM	CEB	FRL
1	CLTB	E1-2	con	1	2711	110	429	2391	2024
2	CLTB	E1-2	con	2	4316	102	423	2196	2731
3	CLTB	E2-3	con	1	5053	177	727	4803	3481
4	CLTB	E2-3	con	2	5214	149	514	3005	3228
5	CLTB	E3-4	con	1	6944	233	796	6110	4714
6	CLTB	E3-4	con	2	8410	195	743	4571	4989
7	CLTB	E4-5	iso	1	39	10	12	3505	2021
8	CLTB	E4-5	iso	2	403	16	26	2159	1969
9	CLTB	E4-6	iso	1	2083	39	284	223	179
10	CLTB	E4-6	iso	2	2821	67	303	297	347
11	CLTB	E5-6	iso	1	95	8	22	2193	1465
12	CLTB	E5-6	iso	2	314	8	18	1724	1497

2. Our additivie model:

$$\theta_{j,k,i,g} = p_{j,k,g} + t_{i,g} + \varepsilon_{j,k,i,g}, \quad (1)$$

where θ denotes log base 2 of the raw counts, p denotes the technical effect, i.e. biases introduced in by MIP reaction, tag sequence and digital counting, t denotes the tissue-specific baseline of gene expression, ε denotes random errors. The notations: i represents tissues, j represents junction, k represents tag measures (1 or 2) for each junction, g represents genes. We fitted model (1) to the *CLTB* data using median polish, which alternatively removes the row (junction-tag) and column (tissue) medians until the proportional reduction of the sum of absolute residuals is negligible (Tukey 1977).

```
> fit1 <- medpolish(log2(dat.CLTB[, 5:9]), trace.iter = FALSE)
```

from $fit1$, we obtained the following estimates.

a). The $\hat{p}$'s are estimated row effects with one value for each junction-tag:

```
> p.hat <- fit1$row
> data.frame(dat.CLTB[, 1:4], p.hat = round(p.hat, digit = 4))
```

	Gene	Exon_Junction	Junction_Type	TAG	p.hat
1	CLTB	E1-2	con	1	0.2447
2	CLTB	E1-2	con	2	0.5275
3	CLTB	E2-3	con	1	1.1568
4	CLTB	E2-3	con	2	0.8169

```
5  CLTB            E3-4            con   1  1.5534
6  CLTB            E3-4            con   2  1.3485
7  CLTB            E4-5            iso   1 -2.9888
8  CLTB            E4-5            iso   2 -2.3108
9  CLTB            E4-6            iso   1 -1.0254
10 CLTB            E4-6            iso   2 -0.2447
11 CLTB            E5-6            iso   1 -3.3108
12 CLTB            E5-6            iso   2 -2.8969
```

b). The $\hat{t}$'s are estimated column effects with one value for each tissue:

```
> t.hat <- fit1$col
> t.hat

        PLA         SKM         STM         CEB         FRL
 0.31205585 -4.57698233 -2.69905843  0.11338141 -0.02412906
```

c). We then obtained our f^* values as the residuals of the additive model:

```
> f.hat <- fit1$residuals
> data.frame(dat.CLTB[, 1:4], round(f.hat, digit = 4))

   Gene Exon_Junction Junction_Type TAG     PLA     SKM     STM     CEB     FRL
1  CLTB          E1-2           con   1 -0.0399  0.2259  0.3115 -0.0224 -0.1253
2  CLTB          E1-2           con   2  0.3482 -0.1658  0.0084 -0.4279  0.0241
3  CLTB          E2-3           con   1 -0.0537  0.0000  0.1603  0.0718 -0.2552
4  CLTB          E2-3           con   2  0.3314  0.0915  0.0000 -0.2649 -0.0241
5  CLTB          E3-4           con   1  0.0083  0.0000 -0.1055  0.0224 -0.2143
6  CLTB          E3-4           con   2  0.4896 -0.0520  0.0000 -0.1914  0.0724
7  CLTB          E4-5           iso   1 -2.9256  0.0000 -1.6149  3.7629  3.1061
8  CLTB          E4-5           iso   2 -0.2344  0.0000 -1.1775  2.3858  2.3904
9  CLTB          E4-6           iso   1  0.8500  0.0000  0.9864 -2.1749 -2.3544
10 CLTB          E4-6           iso   2  0.5069  0.0000  0.2992 -2.5421 -2.1802
11 CLTB          E5-6           iso   1 -1.3192  0.0000 -0.4185  3.4083  2.9638
12 CLTB          E5-6           iso   2 -0.0083 -0.4139 -1.1219  2.6473  2.5811
```

d). And the corresponding median absolute deviation s of f^* for *CLTB*:

```
> s <- mad(f.hat)
> s

[1] 0.3629218
```

3. We took the mean of f^* for the two tags in each junction and tissue. The averaged values for tissue PLA are shown below.

```
> x <- apply(matrix(f.hat[, 1], 2, nrow(f.hat)/2), 2, mean)
> data.frame(dat.CLTB[seq(1, 12, 2), 1:3], PLA = round(x, digit = 4))

  Gene Exon_Junction Junction_Type    PLA
1 CLTB          E1-2           con 0.1542
3 CLTB          E2-3           con 0.1389
```

```
5  CLTB           E3-4           con  0.2489
7  CLTB           E4-5           iso -1.5800
9  CLTB           E4-6           iso  0.6784
11 CLTB           E5-6           iso -0.6638
```

We then calculated the M-score as the averaged values of f^* divided by s.

```
> m.score <- NULL
> for (k in 1:ncol(f.hat)) {
+     x <- apply(matrix(f.hat[, k], 2, nrow(f.hat)/2), 2, mean)
+     m.score <- cbind(m.score, x/s)
+ }
> colnames(m.score) <- colnames(f.hat)
> m.score.CLTB <- data.frame(dat.CLTB[seq(1, 12, 2), 1:3], round(m.score,
+     digit = 4))
> m.score.CLTB
```

```
   Gene Exon_Junction Junction_Type     PLA     SKM     STM     CEB     FRL
1  CLTB           E1-2           con  0.4248  0.0828  0.4406 -0.6204 -0.1394
3  CLTB           E2-3           con  0.3826  0.1260  0.2208 -0.2661 -0.3848
5  CLTB           E3-4           con  0.6859 -0.0716 -0.1453 -0.2328 -0.1955
7  CLTB           E4-5           iso -4.3535  0.0000 -3.8471  8.4711  7.5725
9  CLTB           E4-6           iso  1.8694  0.0000  1.7712 -6.4986 -6.2473
11 CLTB           E5-6           iso -1.8289 -0.5702 -2.1222  8.3428  7.6393
```

The M-scores for CLTB show that there is distinctive tissue specific alternative splicing of exon 5. Exon 5 is preferentially included in smooth muscle derived isoforms (placenta and stomach) and excluded from brain tissue isoforms (cerebellum and frontal lobe).

REFERENCE

Tukey, J. W. (1977). Exploratory Data Analysis, Reading Massachusetts: Addison-Wesley
